# The Effect of Recipient Back-Table Duration on Graft Outcome of Deceased Donor Kidneys: A Single-Center Prospective Cohort Study

**DOI:** 10.3390/jcm12072647

**Published:** 2023-04-02

**Authors:** Julia S. Slagter, Elsaline Rijkse, Roeland F. De Wilde, Roel Haen, Agnieszka Lepiesza, Marie L. Cappelle, Diederik H. J. A. N. Kimenai, Robert C. Minnee

**Affiliations:** Division of HPB and Transplant Surgery, Department of Surgery, Erasmus MC Transplant Institute, 3015 CN Rotterdam, The Netherlands

**Keywords:** kidney transplantation, organ temperature, back-table preparation, delayed graft function

## Abstract

Background: Little is known about the influence of the duration of the kidney back-table preparation period and kidney temperature on graft outcomes after transplantation. The aim of this study is to investigate the back-table duration and its relation to graft outcome and the relation between kidney temperature and graft outcome. Methods: In this prospective cohort study, deceased donor kidney temperature is measured at fixed time points using an infrared thermometer during back-table preparation and transplantation. Additionally, the back-table duration is measured using a timer. Results: Between September 2020 and July 2021, 49 kidneys were prospectively included in this study. Median back-table duration was 33.7 (standard deviation ± 14.1) min and donor kidney temperature increased up to 14.9 °C (±2.8) after 60 min of back-table preparation. Mean implantation time was 24.9 (±7.6) min and kidney temperature increased up to 25.9 °C (±2.4) after 30 min of implantation time. Longer back-table duration was significantly associated with higher rates of delayed graft function (*p* = 0.037). However, this observation did not sustain at 3 and 6-months post-transplantation. No association was found between kidney temperature and graft outcomes. Conclusion: Longer back-table duration is significantly associated with DGF after deceased donor kidney transplantation. No association was observed between kidney temperature and graft outcomes of deceased donor kidneys.

## 1. Introduction

After organ procurement, deceased donor kidney grafts are either stored in 4 °C static cold storage (SCS) or by hypothermic machine perfusion (HMP) in order to reduce metabolism and decrease ischemic injury. After arrival at the recipient transplant center, the graft is inspected and prepared for transplantation, which is the so-called back-table preparation. This period is considered part of the cold ischemia time (CIT), in which the kidney is supposed to remain at 4 °C to optimally reduce metabolism. During transplantation, the graft is exposed to hazardous warm ischemia time (WIT) while the arterial and venous anastomoses are performed in the recipient prior to reperfusion. Some studies have shown that a longer WIT in the recipient leads to an increase in DGF and a decrease in graft and patient survival [1,2]. However, no data on the temperature of the kidney during the anastomosis time were described.

Recent studies show that a longer extraction time during organ procurement of deceased donor kidneys leads to an increase of delayed graft function (DGF) [2,3]. It is likely that the temperature of the donor kidney is higher during extraction due to the perirenal fat surrounding the kidney.

There are very few studies reporting on kidney temperature, especially during back-table preparation. Even though during this procedure the kidney is stored on sludge ice, the kidney will be palpated by the hands of the surgeon and consequently warms up. The kidney is therefore possibly exposed to additional hazardous WIT.

Other studies showed an increase in temperature up to 12 °C during back-table preparation of kidneys donated after brain death (DBD) [4] and an increase up to 13.8 °C in living donor kidneys [5]. However, no data are available on organ temperature of kidney grafts donated after cardiocirculatory death (DCD) or on duration of back-table preparation and graft outcomes.

Therefore, the primary aim of this study is to investigate the association between back-table duration and graft outcome. The secondary aim is to investigate the association between kidney temperature during back-table preparation and transplantation on graft outcome.

## 2. Materials and Methods

### 2.1. Patient and Data Collection

Patients were prospectively included if they were aged 18 years or older and received a deceased donor kidney transplantation. Inclusion started in September 2020. Patients who received a combined organ transplantation or dual kidney transplantation were excluded.

### 2.2. Organ Procurement and Kidney Transplantation Procedure

Organ retrieval was performed in accordance with the local Eurotransplant guidelines. For DCD donors, a period of five minutes of no-touch was maintained before the donor was transported to the operating room. Warm-ischemia time 1 (WIT1) was defined as the time between circulatory arrest and cold perfusion of the aorta in DCD donors. CIT was defined as the time between cold perfusion of the aorta in the donor and the start of the implantation in the recipient, excluding the recipient back-table preparation time to avoid duplication of this period. Warm-ischemia time 2 (WIT2) was defined as the time between the removal of the kidney from SCS at the beginning of the implantation in the recipient and reperfusion of the kidney after vascular anastomoses. Donor nephrectomy time was defined as the time between the cold-flush of the aorta and the removal of the kidney from the donor abdomen.

### 2.3. Measurement of Back-Table Duration and of Kidney Temperature

Back-table duration was measured using a timer. This period was started when the kidney was either removed from SCS or HMP (the latter is the standard of care for deceased donor kidneys procured in the Netherlands) and ended when the kidney was submerged in 4 °C University of Wisconsin solution (UW) and put on SCS before implantation. During back-table preparation, the kidney was kept on a wet gauze on sludge ice.

Graft temperature was measured immediately after removal from SCS or HMP. Subsequently, the temperature was measured every five minutes during the back-table preparation of the kidney, right before flushing of the kidney graft and finally before the kidney was put back on cold storage.

After removal from SCS before implantation, the kidney temperature was measured, and repeatedly every five minutes until reperfusion. Additionally, the temperature was measured just before reperfusion and five minutes after reperfusion. The intra-abdominal temperature was measured just before placement of the kidney into the iliac fossa. Collection of clinical data included donor and recipient characteristics (age, sex, body mass index (BMI), time-on-dialysis), perioperative variables (WIT1, CIT, WIT2), and clinical follow-up.

At the aforementioned time intervals, the kidney temperature was measured using a non-contact infrared thermometer (BTMETER BT-985CAPP; Berrcom Medical Device Co., Ltd., Guangzhou, China). The surface of the kidney graft was identified with a nine-point laser-beam (Figure S1). The mean temperature (2% precision) was calculated from the multiple continuous measurements of the thermometer and was subsequently shown on the optic display.

### 2.4. Outcomes

The primary objective was to investigate the correlation between back-table duration and kidney function, expressed by the eGFR at three-months post-transplantation. Secondary outcomes were the correlation between back-table duration and DGF, the correlation between kidney temperature during back-table preparation and transplantation and 3- and 6-month eGFR and DGF. DGF was defined as the need for dialysis within seven days after transplantation. Primary non-function (PNF) was defined as the absence of any graft function within three months after transplantation.

### 2.5. Ethical Considerations

The study was conducted according to the principles of the Declaration of Helsinki (64th WMA Medical Assembly, Fortaleza Brazil, October 2013). This study was approved by the Erasmus MC Institutional Ethics Review Board (MEC-2020-0667).

### 2.6. Statistics

Categorical variables are presented as a number (percentage). Continuous variables are presented as median (range) or mean with standard deviation, depending on normality. Normality was tested using the Shapiro–Wilks test and visual assessment of histograms. Categorical variables were compared with the Chi-square test. Continuous variables werecompared with the Mann–Whitney-U test. Univariate and multivariate linear regression analysis was used to determine (individual) correlations between variables and 3- and 6-month eGFR. Variables were included in the multivariate linear model if a *p*-value of <0.2 was observed in the univariate linear regression. Multivariate linear regression models were performed using the Enter method. Independent-sample t-test was used to identify the correlation between continuous variables and DGF. Binary logistic regression was used for multivariable analysis for variables that were associated with DGF with a *p*-value of <0.2. Results of the linear regression analyses were presented using a standardized B with 95% confidence intervals (CI). Tests of significance were two-tailed. A *p*-value of ≤0.05 was considered statistically significant. Statistical analysis was performed using the Statistical Package for the Social Sciences (IBM SPSS Statistics, version 25, SPSS inc. Chicago, IL, USA).

## 3. Results

### 3.1. Baseline Characteristics

Between September 2020 and July 2021, a total of 49 kidneys were prospectively included in this study. In all grafts, temperature was measured during back-table preparation. In 28 cases, temperature was also measured during implantation of the kidney.

Median donor age was 60.4 (±10.7) years, 23 donors were male (46.9%), and mean BMI was 26.6 (±3.6) kg/m^2^. The median recipient age was 63 [48.5–68.5], 36 (73.5%) were male, and mean BMI was 28.7 (±5.6) kg/m^2^. Seven recipients were transplanted pre-emptively and median time on dialysis was 35 (±20.8) months. Baseline characteristics are presented in Table 1.

Thirty-two patients received a kidney from a DCD donor (65.3%). The mean WIT1 was 17.2 (±5.9) min in DCD donors. Median CIT was 11.4 [9.58–14.38] h, mean WIT2 was 24.9 (±7.6) min. Mean donor nephrectomy time was 41.0 (±14.3) min and 33/49 (76.3%) were multi-organ donation procedures. Median total operative time was 141 [117–159] min. Arterial reconstruction (end-to-side) was performed during back-table preparation in three cases (6.1%). The median back-table duration was 33.7 (±14.1) min (Table 2).

DGF occurred in 31 recipients (63.3%) of which 21 were DCD kidneys. PNF occurred in three recipients, all of which were DCD kidneys. One of these three patients received a retransplantation. Forty-four patients reached 6-month follow-up. Five patients died. Six-month death-censored graft survival and patient-survival are 94% and 90%, respectively. The mean 3 and 6-month eGFR was 39.4/40.3 mL/min/1.73 m^2^, respectively (Table 2).

### 3.2. Association between Back-Table Duration and Post-Transplant Kidney Function

Univariate linear regression analysis was used to assess the correlation between individual variables and 3 and 6-month eGFR. Three-month eGFR was present in 46 kidney transplant recipients, six-month eGFR was present for 43 kidneys. No association was found between back-table duration and 3 and 6-month eGFR post-transplantation (Table 3).

### 3.3. Association between Back-Table Duration and DGF

Independent sample t-tests were used to assess the correlation between continuous variables and DGF. Pearson chi-square test was used to assess the correlation between categorical variables and DGF. Longer bench duration (95% CI 0.46–16.66, *p* = 0.039) was significantly associated with a higher occurrence of DGF (Table 4A). Logistic regression showed a significant increase in DGF for longer CIT and longer bench duration (Table 4C).

### 3.4. Association between Kidney Temperature and Graft Outcome

Kidney temperature increased up to 14.9 °C (±2.8) after 60 min of back-table preparation and up to 25.9 °C (±2.4) after 30 min of WIT2 during implantation (Table S1 or Table 2, Figure S2 or Figure S3). No associations were found between kidney temperature and graft outcomes.

## 4. Discussion

To our knowledge, this is the first study on the duration of back-table preparation and kidney temperature during transplantation of deceased donor kidneys. Our results show that a longer duration of back-table preparation is associated with inferior short-term outcomes after kidney transplantation. Although no significant association was found between the actual kidney temperature and post-transplant outcomes, we did observe that kidney temperature increases rapidly after removal from SCS or HMP, especially during implantation.

Many factors affect the duration of back-table preparation. First, the experience of the surgeon is of influence and therefore, it is important that surgeons are properly trained in this procedure to ensure optimally short back-table preparation. Speed of the back-table preparation should also be taken into account while training a new surgeon, in addition to merely proper preparation and the avoidance of injury to the organ. Second, the condition of the kidney impacts the back-table duration as kidneys with multiple arteries and veins requiring reconstruction will take longer to prepare, compared to kidneys with single anatomy. Our study only included three kidneys in which arterial reconstruction was performed, thus we did not observe a significantly longer back-table duration when arterial reconstruction was performed, although this would be expected intuitively. Third, the amount of preparation performed by the procurement team impacts the back-table duration. Evidently, preparation of the kidney graft will take longer when there is more remaining tissue surrounding the kidney. This tissue surrounding the kidney may also limit the proper cooling of the kidney during cold storage preservation.

As back-table work is still considered as part of the CIT, the kidney temperature should remain below 5 °C. A better preservation method might be to keep the kidney in a reservoir submerged in cold fluid. Livers are generally prepared using this technique, during which the temperature of the liver graft remains below 5 °C [6]. This preservation method might also reduce the detrimental effect of a prolonged back-table duration for donor kidneys. As a matter of fact, our results could very well be due to this preservation method. It would be interesting to see if the detrimental effects of a prolonged back-table time sustain when the kidney is submerged in cold fluid.

Another method to minimize the CIT could be to perform part of the back-table preparation while on HMP. A small case series on donor livers showed that non-oxygenated CIT could be reduced to over an hour by using this technique [7]. HMP might prevent some of the harmful effects of the back-table period, as (oxygenated) HMP can restore mitochondrial function and ATP-levels [8].

The back-table duration during the procurement surgery is an important variable to address. More preparation during the procurement will most likely lead to a shorter period of back-table preparation at the recipient. It remains to be determined if back-table duration during the procurement surgery also impacts graft outcomes. Currently, no data are available on this subject. This recipient back-table duration is currently not registered in any registries and could possibly be an interesting variable to investigate. It is therefore recommended that donor back-table ischemia time of organs is recorded for future research.

Many studies have reported on the association between longer implantation times and post-transplant outcomes [1,9,10]. This is very likely due to the increase in temperature, during which the kidney again becomes metabolically active, without access to oxygen. Our study showed a temperature increase up to 25.8 °C at 30 min during implantation. This temperature was measured without any efforts to cool the kidney during implantation. Several techniques have been reported to ameliorate the effects of WIT2 and to maintain the kidney temperature below the metabolic threshold of 15 °C [11]. These techniques include surface cooling of the kidney by overlying the kidney [4] or wrapping the kidney in a gauze ‘jacket’ filled with ice-slush [11]. This technique is used in robot-assisted kidney transplantation, due to the longer WIT in these transplantations. A small randomized controlled trial on WIT elimination showed a higher eGFR at 14 days post-transplantation in kidneys that were placed in a gauzed ice jacket during implantation compared to kidneys that were implanted using the standard technique [12]. The same study also showed less acute rejection and DGF in the cooling group [13]. This shows that kidney cooling during the vascular anastomoses might have some protective effect, although more research on this topic is needed.

The temperature we measured during implantation was much higher compared to the temperature measured only in DBD kidneys [4]. A reason for this difference could be that in our study, only the kidney cortex was measured with the infrared thermometer, as compared to the study by Feuillu et al. [4] where a thermosensor was implanted into the kidney parenchyma. This difference in temperature shows that the kidney warms up more quickly on the outside compared to the inside. However, as the glomeruli are located mostly in the kidney cortex, we still consider the measurements executed with the infrared thermometer to be reliable. In addition to this, Feuillu et al. [4] also tried to cool the kidney during implantation using a cold serum.

### Limitations

One of the limitations of this study is the method of temperature measurement of the kidney. During back-table preparation, the kidney is continuously turned by the surgeon in order to be able to prepare both sides of the kidney. As the kidney is placed on ice one side, this side remains colder than the side exposed to the environment, which was also the side where the temperature was measured. This has two consequences, namely the first being that the measured temperature does not represent the overall kidney temperature and the other being that due to the turning of the kidney, the temperature course greatly fluctuates.

Another point to be discussed is the high DGF-rate (63%). This is likely due to the acceptance rate of suboptimal donor kidneys in our hospital, which is a consequence of an increasing number of DCD donors and decrease of DBD donors in the Netherlands. Moreover, donor age of our study population was quite high (60.4 years).

Finally, only 43 kidneys reached the follow-up period of six months. This is a relatively small sample size to support our conclusions. It remains to be determined if the same outcomes appear in a larger sample size. As the overall sample size was small, well-powered studies are required to investigate the association between the back-table duration and graft temperature. Our study already showed an association between longer back-table duration and higher incidence of DGF. Larger studies might also show the association between back-table duration and long-term graft outcomes.

## 5. Conclusions

This study demonstrates an association between longer back-table preparation and higher incidence of DGF in kidney transplantation. In addition, this study also highlights the increase of kidney temperature during back-table work and implantation during transplantation, although no significant associations were found between actual kidney temperature and graft outcomes.

## Figures and Tables

**Table 1:** Baseline characteristics.

Baseline	Mean/Median	Std. Deviation/IQR	Minimum	Maximum	Percentage
Donor Age	60.4	10.7	24	74	
Donor Sex (M/F)					23/26 (46.9%)
Donor BMI	26.5	3.6	20	33	
Recipient Age	63	[48.5–68.5]	24	75	
Recipient Sex (M/F)					36/13 (73.5%)
Recipient BMI	28.7	5.6	18.2	44.3	
Pre-emptive (Yes/No)					7/42 (14.3%)
Time on Dialysis (Months)	35	20.8	3	84	
Total HLA-mismatch	3	[2–4]	0	6	

BMI = body mass index (kg/m^2^), IQR = interquartile range, HLA = human leukocyte antigen.

**Table 2:** Peri- and early post-operative outcomes.

Peri/Post-Operative	Mean/ Mean	Std. Deviation/IQR	Minimum	Maximum	%
DCD/DBD					32/17 (65.3%)
WIT1 (min)	17.2	5.9	6	36	
CIT (min)	684	[574.50–862.5]	260	1508	
WIT2 (min)	24.9	7.6	12	48	
Donor nephrectomy time (min)	41.0	14.3	18	90	
Multi-organ donation					33/49 (67.3%)
Bench duration (min)	33.7	14.1	9	83	
Arterial reconstruction (Yes/No)					3/46 (6.1%)
DGF (Yes/No)					(31/18) 63.3%
PNF (Yes/No)					(3/46) 6.1%
3-month eGFR	38.74	17.7	4	74	
6-month eGFR	40.3	17.8	5	71	
3-month patient survival					47/49 (96%)
6-month patient survival					44/49 (90%)

CIT = cold-ischemia time, DBD = donation after brain death, DCD = donation after cardiocirculatory death, DGF = delayed graft function, eGFR = estimated glomerular filtration rate in mL/min/1.73 m^2^, IQR = interquartile range, PNF = primary non-function, WIT1 = first warm ischemia time (only DCD), WIT2 = second warm ischemia time (anastomosis time).

**Table 3:** Univariate linear regression of eGFR 3 and 6-months post-transplantation.

Univariate 3-Month eGFR	Standard B	95% CI	*p*-Value	Univariate 6-Month eGFR	Standard B	95% CI	*p*-Value
Donor age	−0.209	−0.86–0.14	0.158		−0.326	−1.06–−0.05	0.033
Donor Sex (male)	−0.77	−13.25–7.80	0.605		−0.043	−12.59–9.58	0.785
Donor BMI	0.207	−0.42–2.41	0.163		0.230	−0.36–2.54	0.138
**Recipient age**	**−0.407**	**−0.87–−0.17**	**0.005**		**−0.337**	**−0.81–−0.05**	**0.027**
Recipient sex (male)	−0.072	−14.50–8.91	0.632		0.110	−7.98–16.55	0.484
**Recipient BMI**	**−0.464**	**−2.44–−0.66**	**0.001**		**−0.458**	**−2.52–−0.61**	**0.002**
Time on Dialysis	−0.223	−0.50–0.09	0.166		−0.162	−0.48–0.17	0.339
HLA-mismatch	−0.206	−5.84–1.10	0.176		−0.261	−6.67–0.60	0.1
WIT1	−0.119	−0.77–0.33	0.425		−0.149	−0.86–0.30	0.339
CIT	0.059	−0.19–0.03	0.693		0.191	−0.01–0.04	0.220
WIT2	0.046	−0.58–0.79	0.759		0.067	−0.56–0.86	0.668
Donor nephrectomy time	−0.146	−0.552–0.188	0.326		0.081	−0.338–0.572	0.606
Bench duration	−0.123	−0.52–0.22	0.410		−0.146	−0.57–0.21	0.350

BMI = body mass index, CIT = cold-ischemia time, eGFR = estimated glomerular filtration rate in mL/min/1.73 m^2^, IQR = interquartile range, HLA = human leukocyte antigen, PNF = primary non-function, WIT1 = first warm ischemia time (only DCD), WIT2 = second warm ischemia time (anastomosis time). Bold emphasized statistical significance.

**Table 4:** Variables associated with DGF.

**A.** Independent sample *t*-test for variables associated with DGF				
	**Levene’s Test**	**Sig.**	**95% CI**	***p*-Value**
Donor age	2.570	0.116	−6.93–5.59	0.83
Donor BMI	2.681	0.108	−0.09–1.09	0.938
Recipient age	6.491	0.14	−1.12–16.74	0.084
Recipient BMI	0.710	0.404	−2.36–4.20	0.574
Time on dialysis	0.004	0.948	−9.12–20.08	0.457
WIT 1 (min)	0.890	0.350	−2.66–8.70	0.290
CIT (min)	2.600	0.114	−9.77–266.78	0.068
WIT 2 (min)	0.015	0.903	−1.63–7.41	0.205
Donor nephrectomy time (min)	0.017	0.897	−7.36–9.86	0.771
**Bench duration (min)**	**3.037**	**0.088**	**0.46–16.66**	**0.039**
**B.** Pearson Chi-Square test for categorical variables associates with DGF				
	**Chi-Square**	**Odds Ratio**	**95% CI**	***p*-Value**
**DCD/DBD**	0.221	1.336	0.398–4.483	0.638
**HMP/SCS**	2.416	0.202	0.023–1.794	0.120
**C.** Logistic regression of variables associated with DGF				
	**Beta**	**Exp (B)**	**95% CI**	***p*-Value**
Recipient age	0.046	1.048	0.997–1.101	0.068
**CIT (min)**	**0.004**	**1.004**	**1.000–1.007**	**0.037**
**Bench duration**	**0.067**	**1.069**	**1.004–1.138**	**0.037**

BMI = body mass index, CIT = cold-ischemia time, WIT1 = first warm ischemia time (only DCD), WIT2 = second warm ischemia time (anastomosis time). DCD = donation after cardiocirculatory death, DBD = donation after brain death, HMP = hypothermic machine perfusion, SCS = static cold storage. CIT = cold-ischemia time.

## Data Availability

The data presented in this study are available on request from the corresponding author. The data are not publicly available due to privacy reasons and Dutch legislation.

## Supplementary Materials

The following supporting information can be downloaded at: https://www.mdpi.com/article/10.3390/jcm12072647/s1, Table S1: Temperature course during back-table preparation. SCS = static cold storage. Table S2: Temperature course during implantation. SCS = static cold storage. Figure S1: Laserbeam of thermometer on kidney surface. Figure S2: Temperature course during back-table preparation. Figure S3: Temperature course during implantation. Figure S4: Temperature course during back-table preparation of kidneys with and without DGF. Figure S5: Temperature course during implantation of kidneys with and without DGF.

## References

[1] Heylen L., Naesens M., Jochmans I., Monbaliu D., Lerut E., Claes K., Heye S., Verhamme P., Coosemans W., Bammens B. (2015). The effect of anastomosis time on outcome in recipients of kidneys donated after brain death: A cohort study. Am. J. Transplant..

[2] Heylen L., Pirenne J., Samuel U., Tieken I., Coemans M., Naesens M., Sprangers B., Jochmans I. (2020). Effect of donor nephrectomy time during circulatory-dead donor kidney retrieval on transplant graft failure. Br. J. Surg..

[3] Osband A.J., James N.T., Segev D.L. (2016). Extraction Time of Kidneys From Deceased Donors and Impact on Outcomes. Am. J. Transplant..

[4] Feuillu B., Cormier L., Frimat L., Kessler M., Amrani M., Mangin P., Hubert J. (2003). Kidney warming during transplantation. Transpl. Int..

[5] Kuipers T.G., Hellegering J., El Moumni M., Krikke C., Haveman J.W., Berger S.P., Leuvenink H.G., Pol R.A. (2017). Kidney temperature course during living organ procurement and transplantation. Transpl. Int..

[6] Benjamens S., van den Berg T.A.J., Kuipers T.G.J., Moers C., Berger S.P., Leuvenink H.G.D., Pol R.A. (2020). Kidney temperature during living donor kidney transplantation is associated with short-term measured glomerular filtration rate—A prospective study. Transpl. Int..

[7] Lantinga V.A., Buis C.I., Porte R.J., de Meijer V.E., van Leeuwen O.B. (2022). Reducing cold ischemia time by donor liver “back-table” preparation under continuous oxygenated machine perfusion of the portal vein. Clin. Transplant..

[8] Fuller B.J., Lee C.Y. (2007). Hypothermic perfusion preservation: The future of organ preservation revisited?. Cryobiology..

[9] Hameed A.M., Yuen L., Pang T., Rogers N., Hawthorne W.J., Pleass H.C. (2018). Techniques to Ameliorate the Impact of Second Warm Ischemic Time on Kidney Transplantation Outcomes. Transplant. Proc..

[10] Marzouk K., Lawen J., Alwayn I., Kiberd B.A. (2013). The impact of vascular anastomosis time on early kidney transplant outcomes. Transplant. Res..

[11] Menon M., Sood A., Bhandari M., Kher V., Ghosh P., Abaza R., Jeong W., Ghani K.R., Kumar R.K., Modi P. (2014). Robotic kidney transplantation with regional hypothermia: A step-by-step description of the Vattikuti Urology Institute-Medanta technique (IDEAL phase 2a). Eur. Urol..

[12] Kamińska D., Kościelska-Kasprzak K., Chudoba P., Hałoń A., Mazanowska O., Gomółkiewicz A., Dzięgiel P., Drulis-Fajdasz D., Myszka M., Lepiesza A. (2016). The influence of warm ischemia elimination on kidney injury during transplantation-clinical and molecular study. Sci. Rep..

[13] Kamińska D., Kościelska-Kasprzak K., Chudoba P., Klinger M. (2016). Kidney Injury Due to Warm Ischemia During Transplantation Can Be Reduced. Am. J. Transplant..

