# Supplementary material for: The Effect of Recipient Back-Table Duration on Graft Outcome of Deceased Donor Kidneys: A Single-Center Prospective Cohort Study
Source: J Clin Med. 2023 Apr 2;12(7):2647. doi: 10.3390/jcm12072647 (PMC10094751; doi:10.3390/jcm12072647)
Supplement: Supplementary file 1 [file jcm-12-02647-s001.zip › jcm-2134815-SI.pdf]

Table S1; Temperature course during back-table preparation. SCS = static cold storage

Time	Temperature Mean	Standard deviation
Temperature SCS	3.4	2.1
Temperature 5 min	5.5	2.2
Temperature 10 min	6.7	2.4
Temperature 15 min	7.8	2.4
Temperature 20 min	8.2	2.6
Temperature 25 min	8.3	3.0
Temperature 30 min	8.3	2.2
Temperature 40 min	8.6	3.2
Temperature 50 min	10.0	2.9
Temperature 60 min	14.9	2.8

Table S2; Temperature course during implantation. SCS = static cold storage.

Time	Temperature Mean	Standard deviation
Temperature SCS	4.5	2.9
Temperature 5 min	20.7	3.4
Temperature 10 min	23.7	3.1
Temperature 15 min	24.3	3.4
Temperature 20 min	25.0	2.8
Temperature 25 min	25.1	2.1
Temperature 30 min	25.9	2.4
Temperature 5 min post-reperfusion	32.6	2.3

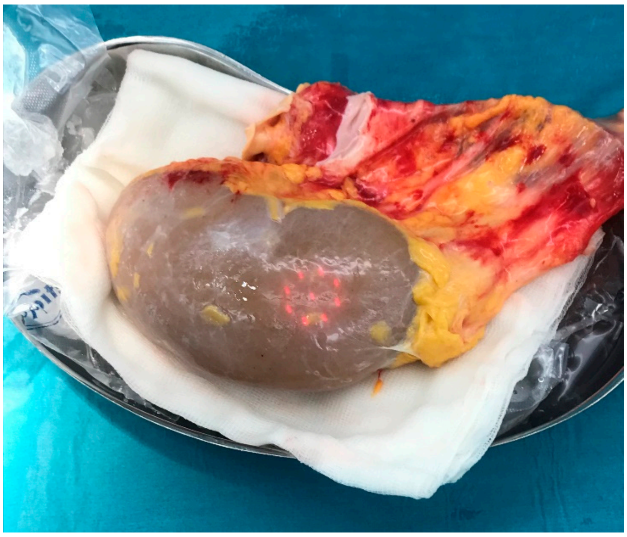

Figure S1: Laserbeam of thermometer on kidney surface.

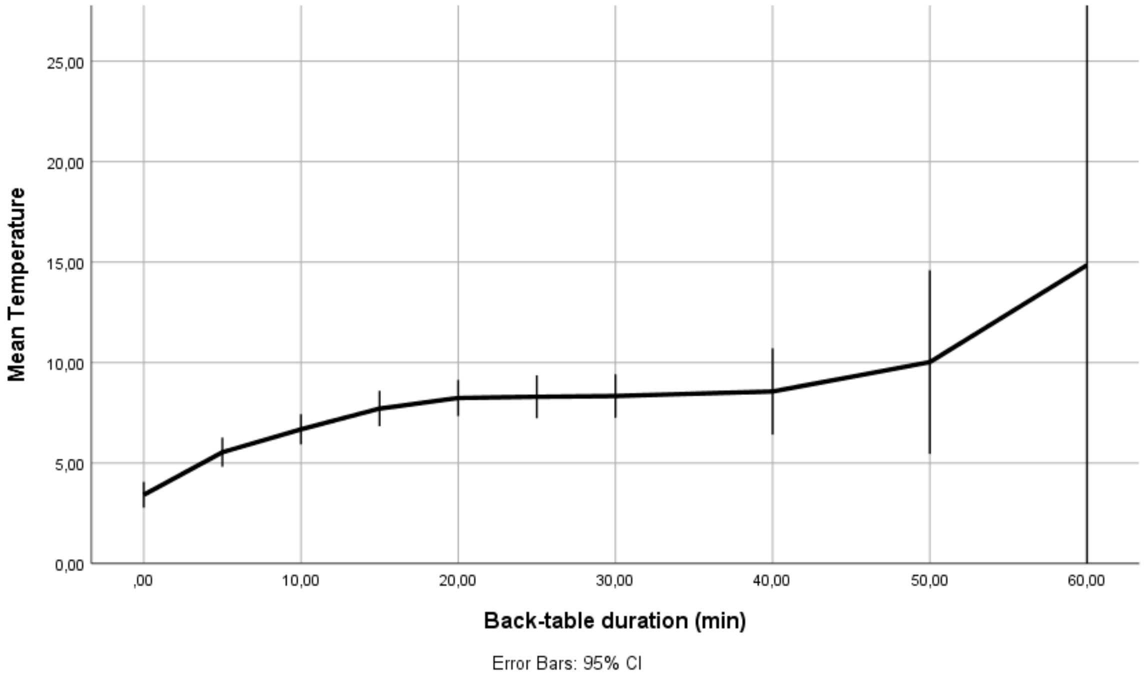

Figure S2; Temperature course during back-table preparation.

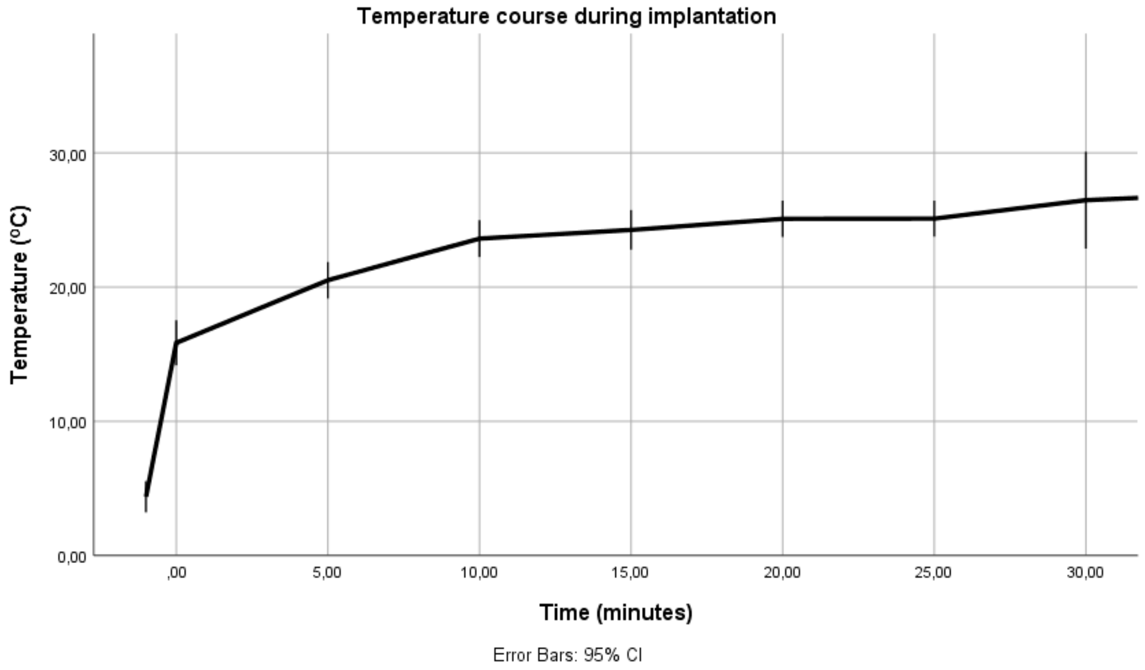

Figure S3; Temperature course during implantation.

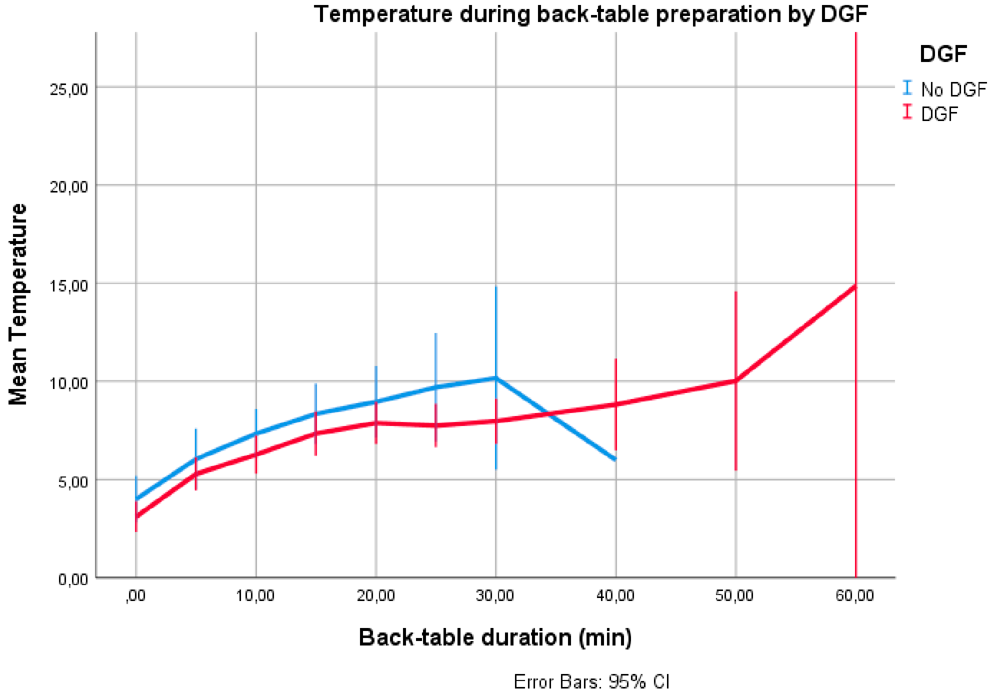

Figure S4; Temperature course during back-table preparation of kidneys with and without DGF.

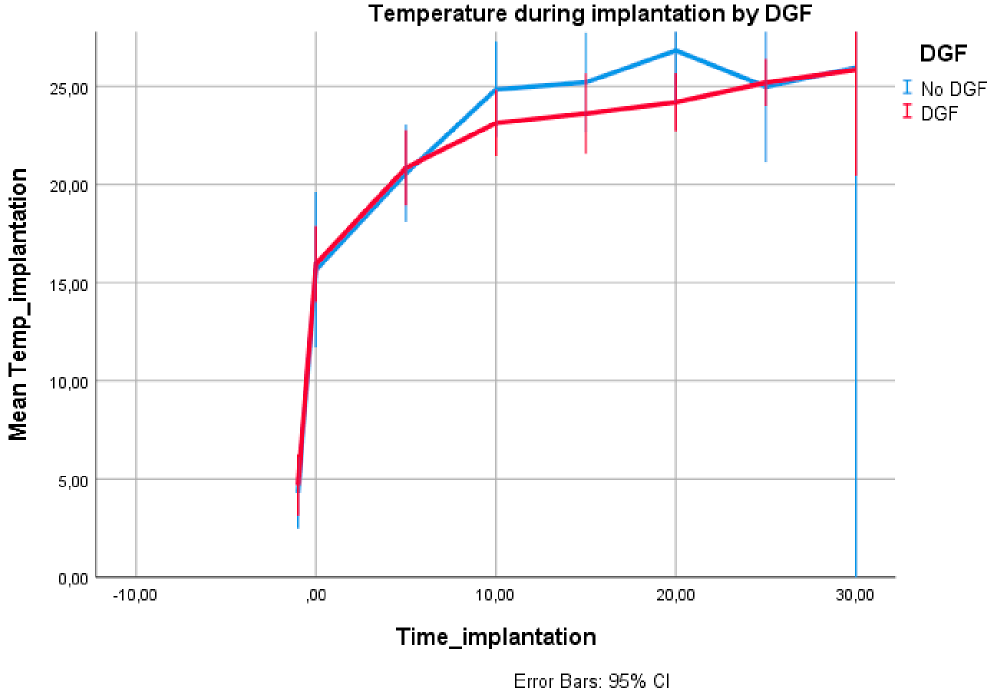

Figure S5; Temperature course during implantation of kidneys with and without DGF.
